# Osteomyelitis pubis caused by *Kingella kingae* in an adult patient: Report of the first case

**DOI:** 10.1186/1471-2334-12-236

**Published:** 2012-10-02

**Authors:** Dunja Wilmes, Patrick Omoumi, Jean Squifflet, Olivier Cornu, Hector Rodriguez-Villalobos, Jean Cyr Yombi

**Affiliations:** 1 Department of Internal Medicine, Cliniques universitaires Saint-Luc, Université Catholique de Louvain, Avenue Hippocrate 10, 1200, Brussels, Belgium; 2 Department of Radiology, Cliniques universitaires Saint-Luc, Université Catholique de Louvain, Avenue Hippocrate 10, 1200, Brussels, Belgium; 3 Department of Gynaecology, Cliniques universitaires Saint-Luc, Université Catholique de Louvain, Avenue Hippocrate 10, 1200, Brussels, Belgium; 4 Department of Orthopaedic Surgery, Cliniques universitaires Saint-Luc, Université Catholique de Louvain, Avenue Hippocrate 10, 1200, Brussels, Belgium; 5 Department of Microbiology, Cliniques universitaires Saint-Luc, Cliniques universitaires Saint-Luc, Université Catholique de Louvain, Avenue Hippocrate 10, 1200, Brussels, Belgium; 6 Department of Internal Medicine and Postoperative Medicine, St Luc University Hospital, Catholic University of Louvain, Avenue Hippocrate 10, 1200, Brussels, Belgium

**Keywords:** Pubic osteomyelitis, *Kingella kingae*, Pubic osteitis

## Abstract

**Background:**

*Kingella kingae* is the second most common pathogen causing paediatric arthritis and is described to be the causative bacteria in some paediatric osteomyelitis. Its microbiological detection is particularly difficult due to its slow growing. To our best knowledge this is the first case description of osteomyelitis pubis caused by this microorganism.

**Case presentation:**

We report the unusual case of pubic osteomyelitis with soft tissue abcess caused by *Kingella kingae* in an adult patient of 66 years with a history of end-stage renal disease and breast carcinoma. Diagnosis was based on imaging and the microorganism was isolated from Computed Tomography-guided aspiration of synovial fluid. The infection resolved completely after twelve weeks of treatment with oral amoxicillin.

**Conclusion:**

This case description highlights the importance in osteoarticular infections of systematic inoculation of synovial liquid in BACTEC vials to optimise the detection of causative organisms, which can necessitate specific treatments.

## Background

Characteristic clinical manifestations of pubic osteomyelitis are severe pelvic pain associated with point tenderness overlying the symphysis pubis and difficulty walking. Differential diagnosis with pubic osteitis, which is more prevalent, can be particularly difficult, because patients present only mild fever, or sometimes are completely apyretic. Pubic osteitis is a non-infectious inflammation of the pubis symphysis, causing varying degrees of lower abdominal and pelvic pain and occurring predominantly in athletes. Only the presence of a microorganism in multiple samples can help to differentiate pubic osteomyelitis of pubic osteitis.

Osteomyelitis of the pubic symphysis is caused by either infectious embolization secondary to bacteraemia or by contiguous spread of infection from soft tissues. Risk factors are trauma, low-grade infection, urological and gynaecological procedures, pelvic malignancies and intravenous drug use [1]. The most frequent pathogens involved in osteomyelitis pubis are *Staphylococcus aureus* and *Pseudomonas aeruginosa.* Polymicrobial infections can occur in infections mainly due to bowel or perineal flora. We summarised in Table 1 the organisms isolated in 117 cases of documented ostemomyelitis of the pubic symphysis reported in the literature.

**Table 1:** Organisms Isolated in 117 cases of microbiologically documented Septic Arthritis of the Symphysis Pubis in the Literature

**Organism**	**No. of Isolates (%)**
***Staphylococcus aureus***	41 (35.04)
***Pseudomonas aeruginosa***	24 (20.51)
**Polymicrobial**	21 (17.94)
Miscellaneous *	10 (8.54)
***Escherichia coli***	5 (4.27)
***Enterococcus*sp.**	5 (4.27)
***Mycobacterium tuberculosis***	4 (3.41)
Streptococcus**	4 (3.41)
Coagulase negative staphylococci ***	3 (2.56)

* Miscellaneous organisms included *Serratia marescens, Brucella melitensis, Bacteroides fragilis, Fusobacterium necrophorum, Citrobacter sp., Peptostreptococcus sp, Lactobacillus, Klebsiella pneumoniae, Corynebacterium accolens, Actinomyces meyeri* (1 patient each).

** Streptococci were two *Streptococcus pneumoniae* and two *Streptococcus agalictiae.*

*** One *Staphylococcus saprophyticus* and two *Staphylococcus epidermidis.*

Since the introduction of the conjugate *Haemophilus influenzae* vaccine, *Kingella kingae* (*K. kingae*) accounts for the second most frequent pathogen of pediatric arthritis, just after *Staphylococcus aureus*[2]. The increasing number of reported cases for this fastidious pathogen is due in part to the improvement of its detection by systematic inoculation of exudates into aerobic BACTEC vials and improved recognition by using PCR assays. In a recently published article, investigators studied characteristics of *K. kingae* osteoarticular infections in children less than four years. This study confirmed, by toxin-specific PCR assays that in infants aged between 6 and 48 months it has become the major bacterial cause of osteoarticular infection. Interestingly, all of their fluid or bone aspirate samples remained negative at gram staining and culture [3].

Unlike paediatric patients, adult patients with invasive *K. kingae* infections present almost exclusively with predisposing medical factors. Previously described predisposing factors are: acquired immunodeficiency syndrome, systemic lupus erythematosus, liver cirrhosis, rheumatoid arthritis, diabetes mellitus, end-stage renal disease, sickle cell anemia, renal transplants, solid tumours, cardiac valvular pathology or haematological malignancies.

In adults, *K. kingae* classically causes endocarditis [4,5], bacteremias [6,7] and spondylodiscitis [8,9]. Descriptions of sacroiliitis [10], pericarditis [11], urinary tract infections [12], lower-respiratory-tract infections [13] and arthritis [4,14] do exist, but remain exceptional. We did not find any case report of osteomyelitis pubis caused by *K. kingae,* neither in adults nor in children.

## Case presentation

A 66-year-old woman of Greek origin was referred by oncologists to our department of infectious disease for pyrexia and pelvic complaints. The patient reported a growing pain localised in the right pubis for 3 weeks. The symptoms were exacerbated by physical exercise and woke her up at night. There was no history of previous trauma. She also presented with low-grade fever (38,2°C), occasionally accompanied by chills. Technetium-99m methyl diphosphonate bone scan showed uptake on right pelvis. She had no recent history of neither urological nor gynaecological interventions.

Her medical history included end-stage renal disease, hypertension, narrow lumbar spinal canal and bilateral breast cancer, treated by surgical resection, radiotherapy and hormonotherapy (Tamoxifen and then Letrozole, stopped in January 2010). The patient had been followed regularly in our Oncology department for over 10 years and her cancer was in complete remission. Her current treatment consisted in lisinopril, simvastatin, cholecalciferol, calcifediol and calcium carbonate.

At the outpatient clinic, the patient was apyretic. Clinical exam showed difficulty walking and a wide-based waddling gait. Reflexes were present symmetrically at both lower limbs and no muscle weakness was objectivised. Heart murmur on Erb site was noted. There were no further abnormal findings.

Blood sample showed a mildly elevated C-reactive protein at 3,3 mg/dL (normal value < 1 mg/dl), elevation of creatinin at 5,95 mg/dL (normal range 0,6-1,3 mg/dl) with a calculated glomerular filtration rate at 8 ml/min/m^2^. Haemoglobin level was low at 10,2 g/dL (normal range 12,0-16,0 g/dl), in association with a high ferritin level, suggesting an inflammatory origin of this normocytic anaemia. Platelet count was raised at 508 000/μl (normal range 150 000–350 000/μl). White blood cell count was normal. Urinary sediment was bland. Two sets of blood cultures and urinary culture were taken the same day and remained negative.

**Figure 1:**
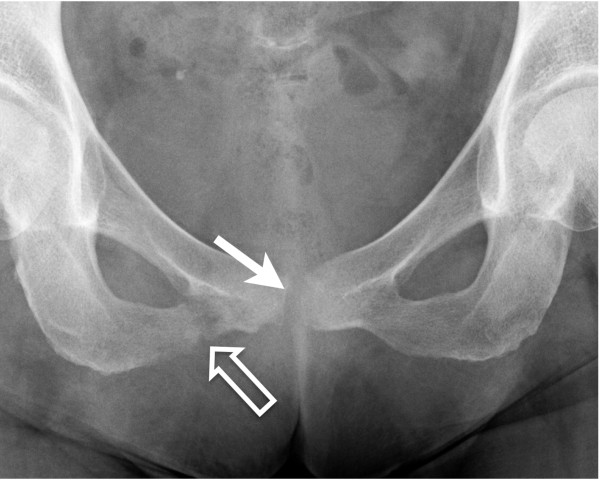
Anteroposterior radiograph of the pelvis shows a fracture of the inferior pubis ramus (open arrow) and irregularity of the symphisis pubis (arrow).

**Figure 2:**
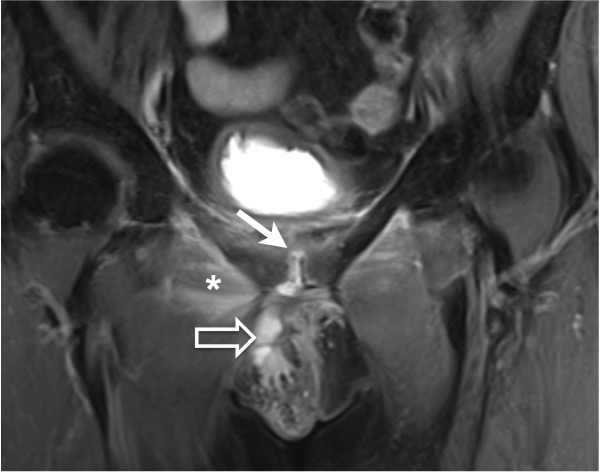
**Fat suppressed proton-density-weighted MRI sequence in the coronal plane shows hypersignal intensity on the pubic symphysis suggesting synovitis (arrow).** These signal abnormalities seemed to be continuous with a voluminous abcess measuring 48 x 22 x 8 mm extending to the external genitalia (open arrow). Edematous infiltration of the right adductor (asterisk) is also shown.

Chest X-Ray was normal. Echocardiography found a little mitral and aortic insufficiency but no evidence of endocarditis. Anteroposterior radiograph of the pelvis showed a fracture of the inferior pubis ramus and irregularity of the symphysis pubis (Figure 1). Magnetic Resonance Imaging (MRI) showed high signal intensity on T2 weighted sequences suggesting pubic symphysitis (Figure 2). These abnormalities seemed to be continuous with a voluminous abscess (48 x 22 x 8 mm) extending to the external genitalia. Furthermore important oedematous infiltration of the right adductor and of the right and left obturator internus and externus was revealed.

**Figure 3:**
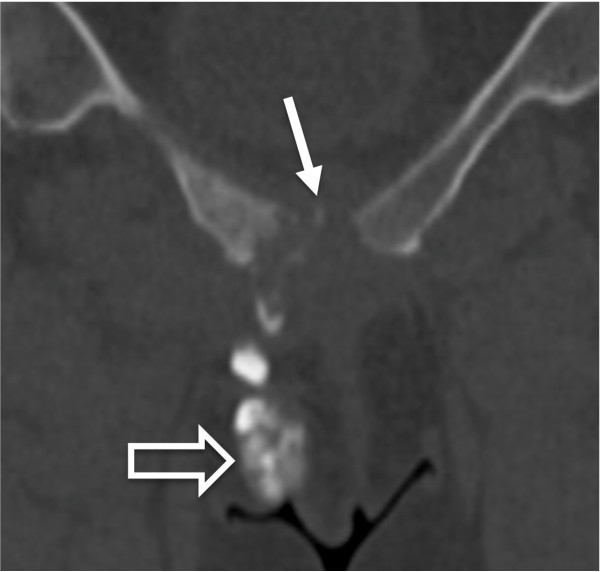
Coronal reformats of computed tomography examination, obtained after direct injection of contrast material into the pubic symphysis (arrow), shows continuity of the arthritis with the collection prolonging to the labia (open arrow).

We decided to assess the extension of the pelvic abnormalities (by injecting contrast material) and to perform an aspiration of the symphysitis for bacterial analysis under Computed Tomography (CT) guidance. We confirmed continuity of the arthritis with the collection, which prolonged to the fracture on one side and to the major labia on the other side (Figure 3). Detailed gynaecologic examination showed swelling of the right major labia, but did not find any entry point for infection nor any fistula.

The culture of the aspirate inoculated in BACTEC aerobe and anaerobe vials, yielded *Kingella kingae (K. kingae)*. Antibiotic susceptibility testing of the isolate showed no resistance. We concluded to pubic osteomyelitis caused by *K. kingae* without any clear entry site. Treatment was started with Amoxicillin 3 grams. We started directly by an oral treatment because the patient did not want a hospitalisation and because sensitivity of *K. kingae* to antibiotics is known to be very good. Furthermore we considered this symphisitis as a chronic osteorticular infection and treated it during 12 weeks. Symptoms disappeared quickly after initiating treatment. A second MRI performed three month later, showed vanishing of the collection, as well as of the symphysitis and a clear regression of the muscle signal abnormalities.

## Discussion

Our patient had no history of neither gynaecological nor urological intervention. Her history was marked by end-stage renal disease (ESRD) and breast carcinoma. ESRD is known to be a risk factor for invasive *K. kingae* infection in adults. A possible explanation could be here a transient *K. kingae* bacteremia, which infected the pubic symphysis. Due to the delay before diagnosis and treatment, an important soft tissue abscess complicated this osteomyelitis

*K. kingae* is part of the normal oropharyngeal flora and has been recognized as an emerging pathogen of paediatric invasive infections. This bacteria requires at least 4 days of incubation to grow. Therefore its detection from purulent specimens is difficult and results in a lack of growth in many instances.The sensitivity of cultures can be improved by inoculating clinical specimens into blood culture vials (automated culturing systems) [15]. Some authors showed that PCR assays increase the sensitivity, particularly if patients are already treated by antibiotics or in pediatric osteoarticular infections [3,11].

In the present case, the isolation of the organism was possible thanks to the inoculation of the CT-guided aspiration fluid into aerobic BACTEC blood culture vials. This was also the reason, why we did not perform PCR assay. All blood and urinary cultures remained negative after 10 days of incubation. Blood cultures in patients with *K. kingae* osteoarthritis are seldom positive [15].

Treatment of osteomyelitis pubis by pyogenic bacterias in the adult is mainly based on surgery. Large debridement of infected and necrotic tissues is recommended and has to be completed by a long course of antimicrobial treatment. However, no clear treatment guidelines in *K. kingae* osteomyelitis are currently available. Invasive surgical procedures seem not to be necessary in most cases, due to the often spectacular response to simple antibiotherapy in arthritis and in discitis [2]. We opted for a treatment by high doses of amoxicillin for a duration of 3 months without surgical intervention, with a good clinical and radiographic response.

## Conclusion

In conclusion, the incidence of infections due to *K. kingae* has been reported with increasing frequency in recent years. However, to our knowledge, no case of osteomyelitis pubis caused by this organism in adults has been described. In our patient, aspiration of the exudate proved infectious inflammation of this amphiarthrodial joint. This case highlights the importance of the systematic inoculation of sterile clinical samples in BACTEC vials, because of the possibility of infection by fastidious bacterias.

## Consent

Written informed consent was obtained from the patient for publication of this Case report and any accompanying images. A copy of the written consent is available for review by the Series Editor of this journal.

## Competing interests

The authors declare that they have no competing interests.

## Authors’ contributions

DW: Manuscript redaction. JCY: Management of the case, manuscript redaction and correction. JS, OC: Clinical management of the case and relecture of the manuscript. PO: Manuscript correction, redaction of the comment of the illustrations. HRV: Manuscript redaction and management of microbiological samples of the patient. All authors read and approved the final manuscript.

## Pre-publication history

The pre-publication history for this paper can be accessed here:

http://www.biomedcentral.com/1471-2334/12/236/prepub
